# Restfulness from sleep and subsequent cardiovascular disease in the general population

**DOI:** 10.1038/s41598-020-76669-z

**Published:** 2020-11-12

**Authors:** Hidehiro Kaneko, Hidetaka Itoh, Hiroyuki Kiriyama, Tatsuya Kamon, Katsuhito Fujiu, Kojiro Morita, Nobuaki Michihata, Taisuke Jo, Norifumi Takeda, Hiroyuki Morita, Hideo Yasunaga, Issei Komuro

**Affiliations:** 1 The Department of Cardiovascular Medicine, The University of Tokyo Hospital, The University of Tokyo, 7-3-1, Hongo, Bunkyo-ku, Tokyo, 113-8655 Japan; 2 The Department of Advanced Cardiology, The University of Tokyo, Tokyo, Japan; 3 The Department of Clinical Epidemiology and Health Economics, School of Public Health, The University of Tokyo, Tokyo, Japan; 4 The Department of Health Services Research, Faculty of Medicine, University of Tsukuba, Tsukuba, Japan; 5 The Department of Health Services Research, The University of Tokyo, Tokyo, Japan

**Keywords:** Medical research, Epidemiology, Risk factors, Cardiology

## Abstract

We aimed to clarify the association between restfulness from sleep and subsequent risk of cardiovascular disease (CVD). Medical records of 1,980,476 individuals with neither prior history of CVD nor sleep disorders were extracted from the Japan Medical Data Center. Restfulness from sleep was subjectively assessed using information from the questionnaires at initial health check-ups. The mean age was 45 ± 11 years and 1,184,937 individuals were men. Overall, 1,197,720 individuals (60.5%) reported having good restfulness from sleep. The mean follow-up period was 1122 ± 827 days. Myocardial infarction, angina pectoris, stroke, heart failure, and atrial fibrillation occurred in 3673 (0.2%), 30,241 (1.5%), 13,546 (0.7%), 28,296 (1.4%), and 8116 (0.4%) individuals, respectively. Multivariable Cox regression analyses including age, sex, and other CVD risk factors after multiple imputation for missing values showed that good restfulness from sleep was associated with lower incidence of myocardial infarction (hazard ratio [HR] 0.89, 95% confidence interval [CI] 0.83–0.95), angina pectoris (HR 0.85, 95% CI 0.83–0.87), stroke (HR 0.85, 95% CI 0.82–0.88), heart failure (HR 0.86, 95% CI 0.84–0.88), and atrial fibrillation (HR 0.93, 95% CI 0.89–0.97). The association of restfulness from sleep with CVD events was pronounced in subjects with younger age and female sex. In conclusion, good restfulness from sleep may be associated with the lower risk of myocardial infarction, angina pectoris, stroke, heart failure, and atrial fibrillation. Further studies are required to clarify the underlying mechanism and to develop a novel preventive approach for CVD from the perspective of sleep.

## Introduction

Sleep is not only considered a resting and relaxing physiological state, but it is also increasingly recognized as an essential indicator of health status. Cardiovascular disease (CVD) is a major cause of morbidity and death^1–3^, and prevention of CVD is an essential field in cardiovascular medicine^4,5^. Sleep is reported to be related to the cardiovascular health^6, 7^ and is currently recognized as an important element in the field of preventive cardiology as well^8^.

Many studies have examined the association between sleep duration and clinical outcomes including CVD incidence^7,9–18^. In addition to the traditional CVD risk factors, both short and long durations of sleep are also reported to be associated with an elevated risk of CVD and all-cause mortality^9–11^. A meta-analysis of prospective studies showed that short sleep duration (relative risk 1.48) and long sleep duration (relative risk 1.38) were both associated with an elevated risk of coronary heart disease^9^.

However, several studies have demonstrated conflicting results regarding the association between sleep duration and clinical outcomes. For example, a prospective cohort study of Taiwanese adults showed that short sleep duration increased the risk of coronary heart disease, whereas long sleep duration did not^14^. The MORGEN study by Hoevenaar-Blom et al. also reported that short sleep duration increased the risk of CVD and the incidence of coronary heart disease^16^. In contrast, a cohort study of a general population in Japan showed that it was not short sleep duration, but long sleep duration, that might increase the risk of CVD-related mortality^15^. A recent meta-analysis also showed that longer sleep duration was associated with all-cause mortality and CVD incidence^7^.

The problem with research on this topic might be the reliability of self-reported sleep duration. Self-reported sleep durations generally indicate total time spent in bed, which is different from the duration of actual physiological sleep. Lauderdale et al. reported that objective sleep duration evaluated using actigraphy was shorter than the self-reported sleep duration^19^. Furthermore, the optimal sleep time for an individual may depend on genetic factors^20^, and appropriate sleep duration varies depending on the individual. Therefore, risk stratification using sleep duration alone has various limitations.

Sleep quality is another important factor in the assessment of sleep. Various studies demonstrated that sleep quality could be also associated with cardiovascular health^21–23^. Several studies have demonstrated that poor subjective sleep quality was independently associated with higher all-cause mortality in older men^23,24^. It has recently been proposed that sleep quality might be more important than sleep duration^25^. Therefore, the importance of comprehensive assessment for restfulness from sleep using both sleep duration and quality is currently recognized. In Japan, the information on restfulness from sleep is routinely obtained using the standard questionnaire in Specific Health Checkups which is the annual health screening and promotion service organized by the Japanese Ministry of Health, Labour and Welfare (MHLW)^26^. In this study, we sought to clarify the association between restfulness from sleep and incident CVD among the general population without prevalent history of CVD using a nationwide epidemiological database.

## Materials and methods

### Study design and data source

This study is a retrospective observational analysis using the health claims database of the Japan Medical Data Center (JMDC; Tokyo, Japan) between January 2005 and August 2018^27–32^. The JMDC contracts with more than 60 insurers and includes data for health insurance claims on more than 5 million registered individuals. Most insured individuals in the JMDC database are employees of relatively large Japanese companies. The JMDC database contains the annual health check-up information of study participants, which includes laboratory data as well as data from questionnaires regarding medical history and medication status. Data on clinical follow-ups obtained by claim records are also included in the JMDC database. Incidence of CVD, including myocardial infarction, angina pectoris, stroke, heart failure, and atrial fibrillation, was evaluated using the International Classification of Disease, 10th Revision (ICD-10) diagnosis codes recorded in the claim records of each individual^33^. We used following ICD-10 diagnosis codes in this study (myocardial infarction: I210, I211, I212, I213, I214, I219, angina pectoris: I200, I201, I208, I209, stroke: I630, I631, I632, I633, I634, I635, I636, I638, I639, I600, I601, I602, I603, I604, I605, I606, I607, I608, I609, I610, I611, I613, I614, I615, I616, I619, I629, heart failure: I500, I501, I509, I110, and atrial fibrillation: I480, I481, I482, I483, I484, I489).

### Ethics

We conducted this study in accordance with the principles of the Declaration of Helsinki. The requirement for informed consent was waived because all data in the JMDC database were anonymized and de-identified. All data were compliant with the International Conference on Harmonization guidelines^34^. Institutional Review Board of the University of Tokyo approved this study (2018-10862). Institutional Review Board of the University of Tokyo also provided waiver for informed consent. We performed this study in accordance with relevant guidelines/regulations.

### Restfulness from sleep

We obtained information regarding restfulness from sleep from questionnaires in the individuals’ initial health check-up (specific health check-up) records. The questionnaire presented at these health check-ups was almost uniform because a regular health check-up is mandatorily performed under the jurisdiction of the Ministry of Health, Labor, and Welfare for most Japanese employees using a standardized format and protocol. If a study participant answered “YES” to the question, “Do you have a good rest with sleep?” then this study participant was categorized as having good restfulness from sleep. If a study participant answered “NO” to this question, then this study participant was categorized as having poor restfulness from sleep.

### Definition

Obesity was defined as body mass index of ≥ 25 kg/m^2^. High waist circumference was defined as ≥ 85 cm for men and ≥ 90 cm for women^35^. Hypertension was defined as systolic blood pressure of ≥ 140 mmHg, diastolic blood pressure of ≥ 90 mmHg, or use of anti-hypertensive medications. Diabetes mellitus was defined as a blood glucose level of ≥ 126 mg/dL, or use of antidiabetic medications including insulin. Dyslipidemia was defined as low-density lipoprotein cholesterol level of ≥ 140 mg/dL, high-density lipoprotein cholesterol level of < 40 mg/dL, triglyceride level of ≥ 150 mg/dL, or use of lipid-lowering medications.

### Statistical procedures

Categorical and continuous data of the baseline characteristics are presented as numbers (%) and means (standard deviation). The chi-square test was used to compare the categorical variables. The unpaired t-test was used to compare continuous variables. We performed the multivariable Cox regression analysis (complete case analysis) to identify prognostic determinants for subsequent CVD events. To account for the missing data values shown in Table 1, we also used multiple imputation as a statistical procedure as previously described^36^. Multiple imputation is a statistical procedure to avert bias caused by missing data by creating multiple filling-in patterns to replace missing values with other plausible values. It is also recognized as an alternative approach for analyzing incomplete data^37^. In this study, we replaced each missing value with a set of substituted plausible values by creating 20 filled-in complete data sets using a multiple imputation with the chained equation method^38^. Covariates included in the multivariable Cox regression analysis were used in the multiple imputation process. Hazard ratio (HR) and standard errors were obtained using Rubin’s rules. As subgroup analyses, we divided our study population by age (≥ 50 years, < 50 years), sex (men, women), and obesity (obese, non-obese), and conducted the multivariable Cox regression analysis in each subgroup. The P values for interactions between groups were calculated. A probability value of < 0.05 was considered statistically significant. In the analysis for interactions, a probability value of < 0.10 was considered statistically significant. We performed all statistical analyses using SPSS software (version 25, SPSS Inc., Chicago, IL, USA) and Stata software (version 16, StataCorp LLC, College Station, TX, USA).

**Table 1:** Characteristics of study population.

Variables	Missing	Restfulness from sleep poor (n = 782,756)	Restfulness from sleep good (n = 1,197,720)	p value
**Age, years**	0	43.8 (10.5)	45.1 (11.4)	< 0.001
20–29 years	0	87,341 (11.2)	133,540 (11.1)	–
30–39 years	0	144,232 (18.4)	193,026 (16.1)	–
40–49 years	0	316,094 (40.4)	449,969 (37.6)	–
50–59 years	0	182,112 (23.3)	277,717 (23.2)	–
60 years	0	52,977 (6.8)	143,468 (12.0)	–
Male sex	0	465,284 (59.4)	719,653 (60.1)	< 0.001
Body mass index, kg/m^2^	78,592	22.8 (3.8)	22.7 (3.5)	< 0.001
Obesity	78,592	177,054 (24.2)	259,942 (22.2)	< 0.001
Waist circumference, cm	237,629	81.0 (10.2)	80.8 (9.6)	< 0.001
High waist circumference	237,629	188,486 (28.1)	290,843 (27.1)	< 0.001
Hypertension	82,872	119,818 (16.4)	210,562 (18.1)	< 0.001
Systolic blood pressure, mmHg	79,272	118.6 (16.2)	119.3 (16.3)	< 0.001
Diastolic blood pressure, mmHg	79,272	73.0 (11.9)	73.5 (11.8)	< 0.001
Diabetes mellitus	474,778	25,992 (4.6)	44,401 (4.7)	< 0.001
Dyslipidemia	134,529	275,343 (38.7)	450,134 (39.7)	< 0.001
Cigarette smoking	3,198	228,398 (29.2)	314,174 (26.3)	< 0.001
Alcohol drinking	20,362	164,147 (21.3)	289,562 (24.4)	< 0.001
**Laboratory data**				
Glucose, mg/dL	479,872	94.1 (17.7)	94.5 (17.0)	< 0.001
HbA1c, %	407,402	5.5 (0.6)	5.5 (0.6)	< 0.001
Low-density lipoprotein cholesterol, mg/dL	131,582	119.6 (31.6)	120.1 (31.5)	< 0.001
High-density lipoprotein cholesterol, mg/dL	127,176	63.3 (16.5)	63.6 (16.6)	< 0.001
Triglycerides, g/dL	127,631	105.6 (87.3)	106.2 (85.0)	< 0.001

Data are expressed as mean (standard deviation) or number (percentage).

## Results

### Study population

We analyzed the data of 2,218,206 individuals who were enrolled in the JMDC database between January 2005 and August 2018 and whose baseline health check-up data (including data on sleep) were available. Exclusion criteria were as follows: (1) age < 20 years (n = 19,948); (2) prior history of myocardial infarction, angina pectoris, coronary revascularization, heart failure, stroke, atrial fibrillation, or hemodialysis (n = 103,011); and (3) existence of sleep disorders defined as ICD-10 code of G47 (e.g., sleep apnea syndrome and insomnia) (n = 114,771). Ultimately, data from 1,980,476 individuals were analyzed in this study. The mean follow-up period was 1122 ± 827 days.

### Baseline clinical characteristics

Characteristics of the study population are shown in Table 1. Overall, the mean age was 45 ± 11 years, and 1,184,937 individuals (59.8%) were men. Among the total cohort, 1,197,720 individuals (60.5%) reported having good restfulness from sleep. Individuals with good restfulness from sleep were older. Obesity and high waist circumference were more common in individuals with poor restfulness from sleep. Cigarette smoking was less common, whereas alcohol drinking was more common, in individuals with good restfulness from sleep.

### Restfulness from sleep and cardiovascular events

During the follow-up period, myocardial infarction, angina pectoris, stroke, heart failure, and atrial fibrillation occurred in 3673 (0.2%), 30,241 (1.5%), 13,546 (0.7%), 28,296 (1.4%), and 8116 (0.4%) individuals, respectively. Multivariable Cox regression analyses including age, sex, and other CVD risk factors, showed that good restfulness from sleep was associated with a lower incidence of myocardial infarction (HR 0.89, 95% CI 0.82–0.96, p = 0.002), angina pectoris (HR 0.85, 95% CI 0.83–0.87, p < 0.001), stroke (HR 0.86, 95% CI 0.83–0.90, p < 0.001), heart failure (HR 0.86, 95% CI 0.83–0.88, p < 0.001), and atrial fibrillation (HR 0.93, 95% CI 0.88–0.98, p = 0.005), as shown in Table 2.

**Table 2:** Multivariable Cox regression analysis for cardiovascular events.

Variables	Hazard ratio	95% Confidence interval	p value
**Myocardial infarction**			
Restfulness from sleep good	0.89	0.82–0.96	0.002
Age, years	1.06	1.06–1.07	< 0.001
Sex (man)	2.36	2.12–2.63	< 0.001
Obesity	1.29	1.16–1.42	< 0.001
High waist circumference	1.07	0.97–1.18	0.190
Hypertension	1.71	1.58–1.86	< 0.001
Diabetes mellitus	1.67	1.50–1.85	< 0.001
Dyslipidemia	1.63	1.50–1.76	< 0.001
Cigarette smoking	1.87	1.73–2.02	< 0.001
Alcohol drinking	0.66	0.60–0.72	< 0.001
**Angina pectoris**			
Restfulness from sleep good	0.85	0.83–0.87	< 0.001
Age, years	1.05	1.04–1.05	< 0.001
Sex (man)	1.10	1.07–1.14	< 0.001
Obesity	1.11	1.07–1.15	< 0.001
High waist circumference	1.14	1.10–1.18	< 0.001
Hypertension	1.59	1.54–1.63	< 0.001
Diabetes mellitus	1.35	1.29–1.41	< 0.001
Dyslipidemia	1.22	1.19–1.25	< 0.001
Cigarette smoking	1.07	1.03–1.10	< 0.001
Alcohol drinking	0.94	0.92–0.97	< 0.001
**Stroke**			
Restfulness from sleep good	0.86	0.83–0.90	< 0.001
Age, years	1.07	1.07–1.08	< 0.001
Sex (man)	0.99	0.95–1.04	0.779
Obesity	1.03	0.98–1.09	0.277
High waist circumference	1.03	0.98–1.09	0.293
Hypertension	1.87	1.79–1.95	< 0.001
Diabetes mellitus	1.33	1.25–1.42	< 0.001
Dyslipidemia	1.10	1.06–1.14	< 0.001
Cigarette smoking	1.30	1.24–1.35	< 0.001
Alcohol drinking	0.99	0.95–1.03	0.656
**Heart failure**			
Restfulness from sleep good	0.86	0.83–0.88	< 0.001
Age, years	1.05	1.05–1.05	< 0.001
Sex (man)	1.07	1.04–1.11	< 0.001
Obesity	1.22	1.17–1.26	< 0.001
High waist circumference	1.11	1.07–1.16	< 0.001
Hypertension	1.92	1.87–1.98	< 0.001
Diabetes mellitus	1.32	1.27–1.38	< 0.001
Dyslipidemia	1.08	1.05–1.11	< 0.001
Cigarette smoking	1.10	1.07–1.14	< 0.001
Alcohol drinking	0.97	0.94–1.00	0.037
**Atrial fibrillation**			
Restfulness from sleep good	0.93	0.88–0.98	0.005
Age, years	1.08	1.08–1.08	< 0.001
Sex (Man)	2.47	2.30–2.65	< 0.001
Obesity	1.11	1.04–1.19	0.002
High waist circumference	1.31	1.23–1.40	< 0.001
Hypertension	1.58	1.49–1.67	< 0.001
Diabetes mellitus	1.12	1.03–1.21	0.009
Dyslipidemia	0.84	0.80–0.89	< 0.001
Cigarette smoking	0.99	0.94–1.05	0.809
Alcohol drinking	1.18	1.12–1.24	< 0.001

Multivariable Cox regression analysis for cardiovascular events presented that good restfulness from sleep was independently associated with the lower incidence of cardiovascular events including myocardial infarction, angina pectoris, stroke, heart failure, and atrial fibrillation.

Multivariable Cox regression analyses including age, sex, and other CVD risk factors, after multiple imputation for missing values, also showed that good restfulness from sleep was associated with a lower incidence of myocardial infarction (HR 0.89, 95% CI 0.83–0.95, p < 0.001), angina pectoris (HR 0.85, 95% CI 0.83–0.87, p < 0.001), stroke (HR 0.85, 95% CI 0.82–0.88, p < 0.001), heart failure (HR 0.86, 95% CI 0.84–0.88, p < 0.001), and atrial fibrillation (HR 0.93, 95% CI 0.89–0.97, p = 0.002), as shown in Table 3.

**Table 3:** Multivariable Cox regression analysis for cardiovascular events after multiple imputation.

Variables	Hazard ratio	95% Confidence interval	p value
**Myocardial infarction**			
Restfulness from sleep good	0.89	0.83–0.95	< 0.001
Age, years	1.06	1.06–1.07	< 0.001
Sex (man)	2.43	2.20–2.68	< 0.001
Obesity	1.26	1.15–1.38	< 0.001
High waist circumference	1.06	0.97–1.17	0.213
Hypertension	1.71	1.58–1.84	< 0.001
Diabetes mellitus	1.67	1.51–1.85	< 0.001
Dyslipidemia	1.65	1.54–1.78	< 0.001
Cigarette smoking	1.88	1.75–2.01	< 0.001
Alcohol drinking	0.66	0.62–0.72	< 0.001
**Angina pectoris**			
Restfulness from sleep good	0.85	0.83–0.87	< 0.001
Age, years	1.05	1.05–1.05	< 0.001
Sex (man)	1.09	1.06–1.12	< 0.001
Obesity	1.11	1.07–1.15	< 0.001
High waist circumference	1.15	1.11–1.19	< 0.001
Hypertension	1.57	1.53–1.62	< 0.001
Diabetes mellitus	1.34	1.29–1.40	< 0.001
Dyslipidemia	1.21	1.18–1.24	< 0.001
Cigarette smoking	1.04	1.01–1.07	0.003
Alcohol drinking	0.94	0.92–0.97	< 0.001
**Stroke**			
Restfulness from sleep good	0.85	0.82–0.88	< 0.001
Age, years	1.07	1.07–1.08	< 0.001
Sex (man)	0.98	0.94–1.02	0.318
Obesity	1.05	0.99–1.10	0.091
High waist circumference	1.04	0.99–1.09	0.129
Hypertension	1.90	1.82–1.97	< 0.001
Diabetes mellitus	1.35	1.27–1.43	< 0.001
Dyslipidemia	1.10	1.06–1.14	< 0.001
Cigarette smoking	1.27	1.22–1.32	< 0.001
Alcohol drinking	1.01	0.97–1.05	0.676
**Heart failure**			
Restfulness from sleep good	0.86	0.84–0.88	< 0.001
Age, years	1.05	1.05–1.05	< 0.001
Sex (man)	1.04	1.01–1.07	0.004
Obesity	1.19	1.15–1.23	< 0.001
High waist circumference	1.13	1.09–1.17	< 0.001
Hypertension	1.94	1.89–1.99	< 0.001
Diabetes mellitus	1.32	1.27–1.38	< 0.001
Dyslipidemia	1.08	1.06–1.11	< 0.001
Cigarette smoking	1.08	1.05–1.11	< 0.001
Alcohol drinking	0.97	0.94–0.99	0.013
**Atrial fibrillation**			
Restfulness from sleep good	0.93	0.89–0.97	0.002
Age, years	1.08	1.08–1.08	< 0.001
Sex (Man)	2.38	2.24–2.54	< 0.001
Obesity	1.09	1.02–1.16	0.008
High waist circumference	1.35	1.27–1.44	< 0.001
Hypertension	1.57	1.50–1.66	< 0.001
Diabetes mellitus	1.12	1.03–1.21	0.008
Dyslipidemia	0.83	0.79–0.87	< 0.001
Cigarette smoking	0.99	0.94–1.04	0.727
Alcohol drinking	1.16	1.11–1.22	< 0.001

Multivariable Cox regression analysis for cardiovascular events after multiple imputation showed that good restfulness from sleep was independently associated with the lower incidence of cardiovascular events including myocardial infarction, angina pectoris, stroke, heart failure, and atrial fibrillation.

### Subgroup analyses

Results of the subgroup analyses are summarized in Table 4. The association between restfulness from sleep and the incidence of myocardial infarction, angina pectoris, stroke, heart failure, and atrial fibrillation which was seen in the overall population, was observed in each subgroup except for the association of restfulness from sleep with the incidence of myocardial infarction, stroke, and atrial fibrillation among individuals with older age, and the association of restfulness from sleep with the incidence of atrial fibrillation among those with obesity. The association of restfulness from sleep with CVD events was significantly modified by age and sex, and was pronounced in subjects with younger age and female sex. The association of restfulness from sleep with CVD events was not modified by the presence of obesity.

**Table 4:** Association of good restfulness from sleep with the incidence of cardiovascular disease in each subgroup.

	Hazard ratio	95% CI	*P* value	*P* for interaction
**Myocardial infarction**				
≥ 50 years	0.99	0.90–1.09	0.855	0.016
< 50 years	0.86	0.77–0.97	0.010	
**Angina pectoris**				
≥ 50 years	0.91	0.87–0.94	< 0.001	0.001
< 50 years	0.85	0.81–0.88	< 0.001	
**Stroke**				
≥ 50 Years	0.97	0.92–1.02	0.190	0.002
< 50 years	0.85	0.80–0.91	< 0.001	
**Heart failure**				
≥ 50 years	0.91	0.87–0.94	< 0.001	0.045
< 50 years	0.87	0.84–0.91	< 0.001	
**Atrial fibrillation**				
≥ 50 years	1.05	0.98–1.12	0.146	< 0.001
< 50 years	0.91	0.84–0.99	0.032	
**Myocardial infarction**				
Men	0.90	0.83–0.98	0.013	0.070
Women	0.80	0.66–0.96	0.017	
**Angina pectoris**				
Men	0.87	0.84–0.90	< 0.001	< 0.001
Women	0.81	0.78–0.85	< 0.001	
**Stroke**				
Men	0.88	0.84–0.93	< 0.001	< 0.001
Women	0.83	0.77–0.88	< 0.001	
**Heart failure**				
Men	0.88	0.85–0.91	< 0.001	< 0.001
Women	0.81	0.77–0.85	< 0.001	
**Atrial fibrillation**				
Men	0.94	0.89–1.00	0.047	< 0.001
Women	0.86	0.76–0.97	0.014	
**Myocardial infarction**				
Obese	0.89	0.79–1.00	0.043	0.342
Non-obese	0.88	0.80–0.98	0.013	
**Angina pectoris**				
Obese	0.85	0.81–0.89	< 0.001	0.370
Non-obese	0.85	0.82–0.88	< 0.001	
**Stroke**				
Obese	0.91	0.85–0.98	0.012	0.201
Non-obese	0.84	0.81–0.88	< 0.001	
**Heart failure**				
Obese	0.86	0.82–0.90	< 0.001	0.122
Non-obese	0.85	0.83–0.88	< 0.001	
**Atrial fibrillation**				
Obese	0.98	0.90–1.08	0.726	0.275
Non-obese	0.90	0.85–0.96	0.001	

Adjusted with sex, obesity, high waist circumference, hypertension, diabetes mellitus, dyslipidemia, cigarette smoking, and alcohol drinking in the subgroup analyses stratified by age.

Adjusted with age, obesity, high waist circumference, hypertension, diabetes mellitus, dyslipidemia, cigarette smoking, and alcohol drinking in the subgroup analyses stratified by sex.

Adjusted with age, sex, high waist circumference, hypertension, diabetes mellitus, dyslipidemia, cigarette smoking, and alcohol drinking in the subgroup analyses stratified by the presence of obesity.

*CI* confidence interval.

The association between restfulness from sleep and the incidence of cardiovascular disease which was seen in the overall population, was observed in each subgroup except for the association of restfulness from sleep with the incidence of myocardial infarction, stroke, and atrial fibrillation among individuals with older age, and the association of restfulness from sleep with the incidence of atrial fibrillation among those with obesity. The association of restfulness from sleep with CVD events was significantly modified by age and sex, whereas not by the presence of obesity.

## Discussion

This comprehensive analysis of a nationwide epidemiological database included approximately two million individuals with neither prior history of CVD nor sleep disorders. The results demonstrated that approximately 60% of the general population reported having good restfulness from sleep and that good restfulness from sleep was independently associated with a lower risk of subsequent incidence of CVD, including myocardial infarction, angina pectoris, stroke, heart failure, and atrial fibrillation. Our results suggest the potential importance of having good restfulness from sleep in the primary prevention of CVD among the general population.

Although previous research has focused more on sleep duration and its effects on the incidence of clinical outcomes rather than on sleep quality^7, 9–18^, several studies have indicated that sleep quality could also affect clinical outcomes including subsequent CVD. For example, a population-based cohort study in Japan showed that poor self-reported sleep quality might be associated with an elevated risk of all-cause mortality independent of sleep duration^39^. Sleep quality assessed by the Jenkins Sleep Questionnaire showed that sleep disturbance was positively correlated with coronary heart disease^21^. A recent meta-analysis showed that sleep quality was associated with coronary heart disease but made no difference in mortality and other outcomes^7^. A systematic review with meta-analysis by Sofi et al. showed that insomnia was associated with the risk of CVD and CVD-related death^22^. Sleep duration and quality are both important, and therefore, we comprehensively assessed “sleep” using the answer of study subjects to the questionnaire regarding restfulness from sleep in this study.

Most studies regarding sleep and CVD have focused on its association with the incidence of coronary heart disease. Our study gives the first epidemiological data analyzing the relationship between restfulness from sleep and the incidence of various CVDs; this includes not only coronary heart disease but also other major CVDs such as stroke, heart failure, and atrial fibrillation. Furthermore, because sleep disorders are known to increase the risk of CVD^40,41^, we excluded individuals with established diagnoses of sleep disorders (e.g., sleep apnea syndrome and insomnia) from the analysis of this study. Still, having good restfulness from sleep was independently associated with the lower incidence of myocardial infarction, angina pectoris, stroke, heart failure, and atrial fibrillation.

Our study has clinical implications. Approximately 40% of the individuals reported poor restfulness from sleep, and that poor restfulness from sleep might elevate the risk of various CVDs. From this point, intervention for sleep may have great potential in preventive cardiology. It should also be noted that we could stratify the risk of CVD by simply asking individuals a short question about sleep. We believe that asking this simple question could be possible even in busy real-world clinical or public health settings and could provide physicians with important information on future CVD risk.

Results of subgroup analyses are important as well. The association of restfulness from sleep with CVD events was pronounced in subjects with younger age and female sex. Additionally, individuals not having restfulness from sleep were more common in those with younger age. The study population in this study are working for relatively large Japanese companies. Younger subjects serving for such companies in Japan, may be prone to be quite busy, have much mental or physical stress, and irregular lifestyles, which could be related to “poor sleep”. Unfortunately, we did not have enough data assessing this point concretely. Further investigations are required to clarify the mechanism of these results. Considering that the importance of CVD prevention among young adults is increasingly recognized currently, restfulness from sleep could have a potential as a key element for CVD prevention in young generations, particularly younger women.

The next essential issues to be addressed are to clarify if interventions that improve restfulness from sleep is effective in CVD prevention and how to intervene in poor restfulness from sleep. Various psychological, physiological, social, and environmental factors could lead to poor restfulness from sleep, and there may be individuals with latent CVD among those with poor restfulness from sleep. Therefore, root cause investigation for poor restfulness from sleep is indispensable as the first step. The beneficial impact of good restfulness from sleep on subsequent incidence of CVD is seemingly similar regardless of the targeted disease. Therefore, we speculate that good restfulness from sleep might influence primitive development of CVD.

Several possible mechanisms can be suggested to explain the association between restfulness from sleep and the subsequent incidence of CVD. Poor sleep is known to induce leptin/ghrelin imbalance and to impair glycemic control, and these could lead to obese and metabolic disorders^42–46^. Sleep is also an important regulator of circadian rhythm, which is associated with the development of CVD^47^, and individuals with poor sleep quality may have disturbed circadian rhythms. Kondo et al. reported that the cyclic alternating pattern during sleep was associated with heart rate variability, blood pressure, and autonomic activity^48^, which could also affect the development of CVD. Meier-Ewert et al. demonstrated that sleep disorders were associated with elevated serum C-reactive protein levels, suggesting that inadequate sleep may cause chronic inflammation^49^, which plays a pivotal role in the pathology of various CVDs^50,51^. Sleep disturbances were also reported to accelerate subclinical CVD burden including coronary artery calcium, carotid intima media thickness, endothelial dysfunction, and aortic stiffness^52^. Further studies are warranted to elucidate the underlying pathophysiological mechanisms of our results.

There are several limitations in this study that need to be addressed. Although we performed multivariable Cox regression analyses, we could not eliminate the possibility of unmeasured confounders and residual bias. Data on sleep duration were unavailable in our database. Because the population in the JMDC database comprised employed working-age individuals, healthy worker bias should be acknowledged. Further investigations are required to confirm our results and to generalize them with populations of different ethnicities, races, education levels, and incomes. Comparing with other nationwide epidemiological data in Japan^53,54^, the incidence of CVD in our study is reasonable, and therefore, we believe that our data could have reflected real-world clinical practice. However, recorded diagnoses are generally considered less well validated due to the nature of retrospective design and administrative database. Although the mood is closely related to sleep, our database lacked the information on the mood.

In conclusion, this comprehensive analysis of a nationwide epidemiological database suggested that good restfulness from sleep was associated with a lower subsequent incidence of myocardial infarction, angina pectoris, stroke, heart failure, and atrial fibrillation among the general population without prior history of relevant CVD. This exemplifies the potential essential role of sleep in the primary prevention of CVD.
